# Spontaneous massive pectoral hematoma induced by vitamin K antagonist therapy: a case report

**DOI:** 10.11604/pamj.2021.38.324.28454

**Published:** 2021-04-01

**Authors:** Karima Benbouchta, Asmae Mrabet, Ossema Kallel, Noha El Ouafi, Zakaria Bazid

**Affiliations:** 1 Department of Cardiology, Mohammed VI University Hospital of Oujda, Mohammed I University of Oujda, Oujda, Morocco,; 2 Laboratory of Epidemiology, Clinical Research and Public Health, Faculty of Medicine and Pharmacy, Mohammed I University of Oujda, Oujda, Morocco

**Keywords:** Spontaneous hematoma, anticoagulation, vitamin K antagonists, pectoral, case report

## Abstract

Vitamin K antagonists (VKA) based oral anticoagulation, is widely used for the prevention and treatment of thromboembolic disease. The major complication of this therapy is bleeding, and sometimes it can occur in unsuspected areas. Spontaneous pectoral hematoma is one of the rare complications due to over anticoagulation by VKA therapy, with only a few cases reported in the literature. Concomitant use of this therapy with commonly used antibiotic, especially in the elderly with multiple comorbidities, can increase the risk of bleeding. Herein, we report a case of a 72-year-old woman under VKA for the treatment of atrial fibrillation, who presented with a spontaneous massive pectoral hematoma, while using antibiotic to treat a respiratory tract infection, who was successfully managed.

## Introduction

Vitamin K antagonists (VKA) based oral anticoagulation, is widely used for the prevention and treatment of thromboembolic disease. However, those medications may cause serious adverse events such as bleeding or hematomas at various anatomic areas [1], but sometimes they may occur at unsuspected sites. Spontaneous pectoral hematoma (SPH) is a very rare complication due to over anticoagulation by VKA therapy, with only a few cases reported in the literature. Herein, we report a case of a spontaneous massive pectoral hematoma during therapy with VKA for the treatment of atrial fibrillation in an elderly woman.

## Patient and observation

**Figure 1:**
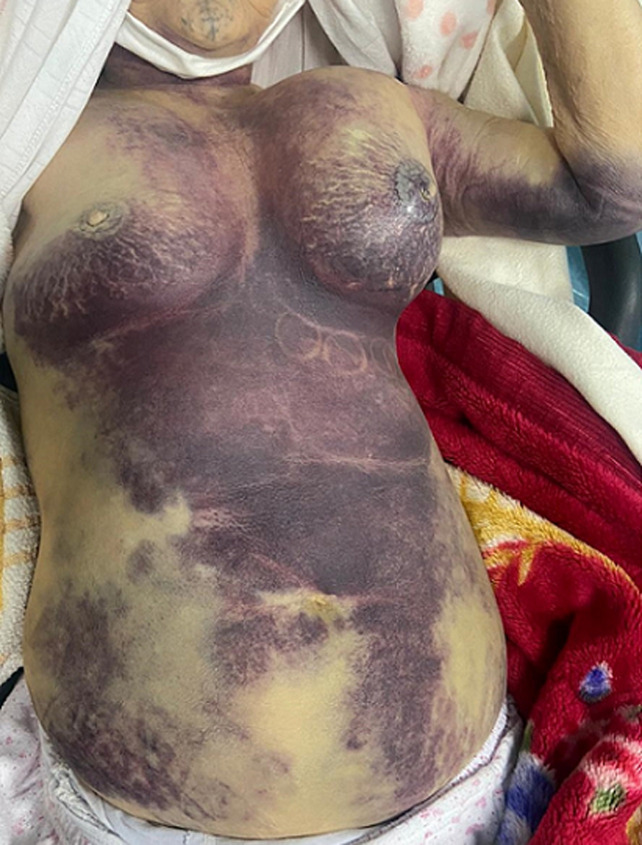
image showing signs of massive pectoral hematoma; important swelling of the left anterior chest wall, with significant ecchymosis extending to the left arm, right breast, and the anterior abdominal wall

A 72-year-old female patient with a history of seven years of atrial fibrillation that received VKA therapy (Acenocoumarol 2mg per day), presented to the emergency department with 3 days history of gradually worsening left breast swelling and bruising. There were no precipitating factors, trauma, or strenuous exertion. Clinical examination revealed a hemodynamically stable patient with anemic conjunctiva. There was a tender swelling in the left breast and the left-sided chest wall with significant ecchymosis extending to the left arm, right breast, and the anterior abdominal wall (Figure 1).

**Figure 2:**
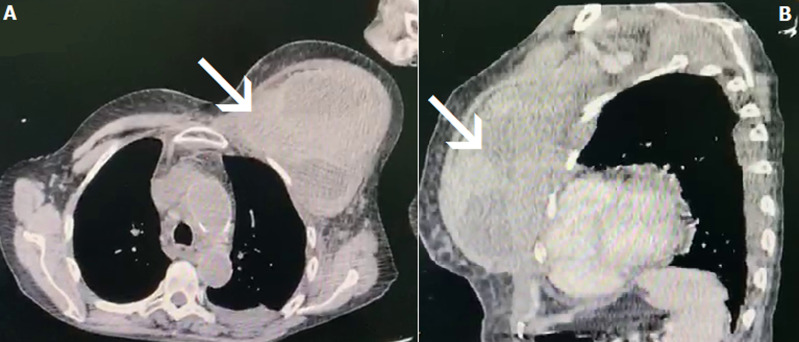
contrast enhanced tomography scan of the chest images; (A) coronal view and (B) sagittal view, showing massive sub-pectoral hematoma (arrow) measuring 153x123 mm without extravasation of contrast medium

Laboratory investigations revealed severe anemia (hemoglobin 5.4 g/dl), a markedly high international normalized ratio of prothrombin (INR > 10), and a renal failure (creatinine 25 mg/l), platelet count and liver function test were within normal ranges. Subsequent history obtained from the family revealed that the patient had been coughing last week, and she was prescribed Amoxicillin Clavulanate 1g thrice daily, to treat a respiratory tract infection five days prior to the onset of symptoms. Immediate suspension of VKA was done, and the patient was transfused by two units of red blood cells (RBC), and fresh frozen plasma (FFP) and she received 10 mg of vitamin K. An urgent contrast enhanced computed tomography (CT) scan of the chest confirmed the presence of a massive sub-pectoral and axillar hematoma measuring 153x123 mm without extravasation of contrast medium (Figure 2).

After a discussion among cardiologists, vascular surgeons, and anesthesiologists, the decision was to continue supportive management and keep the patient under close clinical supervision, as there was no active arterial diathesis in CT scan. The patient received in total seven units of RBC and five units of FFP. After obtaining an INR of 1.5, hematoma evacuation was realized to prevent skin and breast ischemia. During surgery, a large amount of blood clots was evacuated, but no bleeding vessel was found. The incision was closed after a drain was placed, and compression was applied. The patient had an uncomplicated postoperative course, without any signs of recurrent bleeding, and her serial hemoglobin had stabilized without further decrease, and she was discharged one week later.

## Discussion

Anticoagulation therapies, including VKA (Warfarin/Acenocoumarol/Phenprocoumon), are widely used for the preventive and treatment of thromboembolic disease. However, those medications may cause major adverse events such as bleeding or hematoma in many anatomical structures [1]. Spontaneous pectoral hematoma (SPH) is rarely reported in the literature, as a complication of anticoagulation using VKA therapy, especially without preceding trauma or invasive medical procedure [1-6]. Clinical presentation of SPH may be variable, and it depends on the hematoma volume, which can range from a small one without any symptoms, to a large and massive one that may present with hemorrhagic shock and can be life-threatening [3]. The typical presentation reported in the literature of pectoral hematoma is an acute development of swelling in the anterolateral chest wall, with ecchymosis, tenderness, and limitation of arm movements [7]. Ultrasonography and computed tomography (CT) are commonly used methods of definitive diagnosis [4]. Furthermore, contrast-enhanced CT plays an essential role in the management of SPH. It determines whether active bleeding is present or not, and identifies the location of the vascular source of bleeding [2,4]. The management of SPH will vary according to the patient´s status, and it can be conservative or invasive. Supportive therapy is essential in the management of SPH. It includes a cessation of anticoagulation, blood transfusion as needed, and the administration of vitamin K, FFP, and even the prothrombin complex concentrate, in order to reverse the effect of VKA therapy. If the patient is hemodynamically stable, conservative treatment is preferable, with close monitoring of vitals, hemoglobin, and INR [8]. In cases of no improvement after conservative treatment, or when the patient is in hemorrhagic shock, due to uncontrolled bleeding, invasive treatment is indicated, which can be an open surgery or transcatheter arterial embolization [5,9-10]. Hematoma evacuation can also be considered in cases of extensive hematoma with increased risk of local complications such as local compression and infection [4].

Pathogenesis of SPH formation in patients on VKA therapy may be multifactorial. Old age presents the principal factor that increases the risk of major bleeding, due to multiple co-morbid diseases like renal failure, or liver disease, also due to an increase in endothelial vascular fragility [2,11]. Sometimes a minor trauma or only a cough can trigger bleeding by causing capillary tears [4,7,12]. In addition, concomitant use of VKA with some medications, like antiplatelet, nonsteroidal anti-inflammatory drugs, or some antibiotics, and drug-drug interactions, are also reported as risk factors [11]. In our case, various factors had predisposed to spontaneous bleeding and the formation of a pectoral hematoma. The concomitant use of VKA therapy and Amoxicillin Clavulanate had increased the chance for a drug-drug interaction; also the renal failure had potentiated the action of VKA therapy. Even coughing could have triggered bleeding due to a tear in regional blood vessels that became more fragile with advanced age.

## Conclusion

Spontaneous pectoral hematoma presents one of the rare complications reported in the literature resulting from over anticoagulation by VKA therapy. The use of this therapy requires the physicians to be vigilant of this possible side effect and to be cautious while prescribing other medications, and keep in mind that major bleeding may occur at various areas because of drug-drug interactions, especially in the elderly with multiple comorbidities.
